# Potential of a New, Flexible Electrode sEMG System in Detecting Electromyographic Activation in Low Back Muscles during Clinical Tests: A Pilot Study on Wearables for Pain Management

**DOI:** 10.3390/s24144510

**Published:** 2024-07-12

**Authors:** Antoine Frasie, Hugo Massé-Alarie, Mathieu Bielmann, Nicolas Gauthier, Mourad Roudjane, Isabelle Pagé, Benoit Gosselin, Jean-Sébastien Roy, Younes Messaddeq, Laurent J. Bouyer

**Affiliations:** 1 Center for Interdisciplinary Research in Rehabilitation and Social Integration (CIRRIS), Centre Intégré Universitaire de Santé et de Services Sociaux de la Capitale Nationale (CIUSSS-CN), Quebec City, QC G1M 2S8, Canada; antoine.frasie.1@ulaval.ca (A.F.); hugo.masse-alarie@fmed.ulaval.ca (H.M.-A.); mathieu.bielmann.1@ulaval.ca (M.B.); nicolas.gauthier.11@ulaval.ca (N.G.); isabelle.page1@utqr.ca (I.P.); benoit.gosselin@gel.ulaval.ca (B.G.); jean-sebastien.roy@fmed.ulaval.ca (J.-S.R.); younes.messaddeq@copl.ulaval.ca (Y.M.); 2 School of Rehabilitation Sciences, Faculty of Medicine, Université Laval, Quebec City, QC G1M 2X8, Canada; mourad.roudjane.1@ulaval.ca; 3 Department of Computer and Electrical Engineering, Faculty of Science and Engineering, Université Laval, Quebec City, QC G1V 0A6, Canada; 4 Center for Optics, Photonics and Lasers (COPL), Department of Physics, Faculty of Science and Engineering, Université Laval, Quebec City, QC G1V 0A6, Canada

**Keywords:** low back pain, clinical tests, wearable sensors, sEMG, muscle fatigue, sensorimotor control

## Abstract

Background: While low back pain (LBP) is the leading cause of disability worldwide, its clinical objective assessment is currently limited. Part of this syndrome arises from the abnormal sensorimotor control of back muscles, involving increased muscle fatigability (i.e., assessed with the Biering–Sorensen test) and abnormal muscle activation patterns (i.e., the flexion–extension test). Surface electromyography (sEMG) provides objective measures of muscle fatigue development (median frequency drop, MDF) and activation patterns (RMS amplitude change). This study therefore assessed the sensitivity and validity of a novel and flexible sEMG system (NSS) based on PEVA electrodes and potentially embeddable in textiles, as a tool for objective clinical LBP assessment. Methods: Twelve participants wearing NSS and a commercial laboratory sEMG system (CSS) performed two clinical tests used in LBP assessment (Biering–Sorensen and flexion–extension). Erector spinae muscle activity was recorded at T12-L1 and L4-L5. Results: NSS showed sensitivity to sEMG changes associated with fatigue development and muscle activations during flexion–extension movements (*p* < 0.05) that were similar to CSS (*p* > 0.05). Raw signals showed moderate cross-correlations (MDF: 0.60–0.68; RMS: 0.53–0.62). Adding conductive gel to the PEVA electrodes did not influence sEMG signal interpretation (*p* > 0.05). Conclusions: This novel sEMG system is promising for assessing electrophysiological indicators of LBP during clinical tests.

## 1. Introduction

The recent emergence of miniature wireless sensing technologies integrated into smart textiles for recording human physiological signals presents important potential for conducting laboratory-grade recordings in clinical settings during standardized clinical tests and activities of daily living. One of the potential applications of this technology is to assist the challenging management of low back pain.

Low back pain is the leading cause of disability worldwide, with the number of prevalence cases increasing by 60.4% between 1990 and 2020, while the percentage of years lived with low back pain-related disability decreased by 10.5% (1). The associated costs, both direct and indirect, continue to rise, ranging between USD 2.2 billion (in low- and middle-income countries) (2) and USD 134.5 billion (in the USA) (3). This is partly due to the recurrence and chronicization of low back pain [1].

Low back pain is a complex problem, and its origin is multifactorial [2]. Alterations in sensorimotor control are potential contributors [3,4,5,6,7,8,9], characterized by changes in muscle activations, the early development of muscle fatigue, and the distorted input or interpretation of afferent sensory information including pain [10,11]. In people with chronic LBP, motor control exercises aim to target and normalize these alterations to reduce pain and disability [12,13].

Low back pain-related sensorimotor alterations can be assessed in part using clinical tests. For example, the early onset of fatigue in erector spinae muscles can be revealed by reductions in the duration of posture maintenance [14] or force output [15,16] during the Biering–Sorensen test, an isometric endurance test of erector spinae muscles [17]. In addition, altered erector spinae muscle activations can be inferred (visually or by palpation) during standing trunk flexion [18,19]. Altogether, clinical measures of low back pain-related sensorimotor alterations remain global, encompassing factors like endurance time, visual assessments, and palpation assessments [20].

The use of surface electromyography (sEMG) during the above-mentioned clinical tests [20,21,22] would allow for the quantification of motor performance, and could therefore be a useful tool for clinicians for the diagnosis, prognosis, and choice of rehabilitation strategies for individuals with low back pain. For example, a significant drop in the median frequency of erector spinae sEMG power spectrum (MDF) [23] during the Biering–Sorensen test could serve as an indicator of muscle fatigue development before test failure [24]. Furthermore, placing multiple sEMG sensors over lower back muscles could accurately measure fatigue location [24,25]. Finally, recording erector spinae sEMG amplitude (Root Mean Square amplitude, RMS) during a dynamic test such as the standing trunk flexion test [26] (named the flexion–extension test) [26,27,28,29] would assist in identifying muscle activation alteration during different phases of movement more accurately and quantitatively than the current visual analysis conducted in clinical settings [27,28,29].

Currently, the cost, size, stiffness, and complexity of installing/use of commercially available sEMG systems make their use impractical in clinical settings [30,31]. In fact, there is currently no miniature, flexible, low-power, and washable sEMG sensor system available that allows for an integrated operational solution in garment fabrics for recording and analyzing lower back sEMG in clinical settings and during activities of daily living [32,33]. Our interdisciplinary research group, composed of specialists in physics, engineering, and rehabilitation, has recently developed a novel sEMG system (NSS) that can be embedded into garments to assess muscular activity [34,35]. As an initial step in the development of an instrumented shirt for assessing electromyographic alterations associated with low back pain, the sensitivity and validity of the NSS need to be assessed. 

To assess the clinical applicability of the NSS, the first objective of this study was therefore to measure the sensitivity of the NSS for two specific electromyographic indicators during clinical tests: (a) the drop in MDF, as a measure of fatigue development; and (b) changes in RMS levels, as a measure of muscle activation patterns.

To assess the validity of the NSS, the second objective was to evaluate the NSS signal quality by comparing the NSS-measured indicators to those obtained from a commercial laboratory-grade sEMG system (CSS; considered the gold standard) obtained during the same clinical tests. To do so, (a) relative responses (% MDF and RMS ratios), and (b) raw signal (MDF and RMS) over time were specifically examined.

Finally, as a dry electrode-to-skin interface is much more practical for use in non-laboratory settings, the third objective of the study was to assess the impact of conductive gel versus dry skin–electrode interfaces on NSS performance.

## 2. Materials and Methods

### 2.1. Participants

To be included in the study, individuals had to be aged between 18 and 65 years and report no pain, movement limitations, or a history of surgery, fractures, or diseases (e.g., inflammatory conditions) to the lower back [36]. Exclusion criteria were as follows: (1) low back pain within the last twelve weeks that significantly impacted daily life, (2) a self-reported history of scoliosis (as scoliosis could induce asymmetrical muscle activations) (41), and (3) pregnancy or giving birth within the previous 12 months. Participants were recruited through the institutional mailing list of Université Laval. This study was approved by the local ethics committee (CIUSSS-CN #2019-1798).

### 2.2. General Protocol

**Figure 1:**
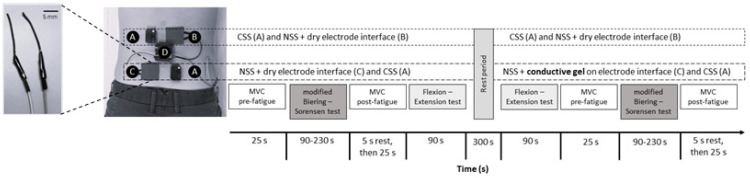
Sensor set up and sequence of the laboratory tests. Commercial sEMG laboratory system (CSS: A) and new sEMG system electrodes (NSS: B and C) both at T12-L1 and L4-L5 spinal levels. The NSS reference electrode was fixed on the right iliac crest, and the process control block on the lumbar spine (D). In the second set of tests, conductive gel was added to the NSS electrode interface at the L4-L5 spinal level (C). Left inset: close-up view of the carbon nanotube–PEVA electrodes of the NSS system.

Participants came to the laboratory for a single session. Upon arrival, the experimenter explained the protocol, obtained written consent, and collected sociodemographic information. Participants were then instrumented with both the NSS and the CSS, according to the configuration shown in Figure 1, with both systems simultaneously recording erector spinae sEMG activity at T12-L1 and L4-L5 levels.

The laboratory session was divided into two sets of four tests. Each set included an initial maximal voluntary contraction (MVC) assessment of erector spinae muscles (pre-fatigue), a modified Biering–Sorensen test, a second MVC assessment (post-fatigue), and a flexion–extension test (Figure 1). The NSS electrode-to-skin interface changed between the two sets at the L4-L5 level: the first set was conducted using a dry electrode/skin interface, while the second set used a conductive gel interface. The CSS kept the same configuration and skin interface during all tests.

### 2.3. Materials

#### 2.3.1. The New sEMG System (NSS)

The NSS is composed of a pair of multi-material polymer fibers [34,35] (3 cm length, 2 cm inter electrode spacing; Figure 1, left inset) serving as the sEMG sensor, and a process control block for the associated electronics (Figure 1, ‘D’). The multi-material polymer fibers are made of a mixture of multi-walled carbon nanotubes (Cheap Tubes, Cambridgeport, VT, USA) with a carbon purity of 95 wt.% (percentage by weight) and poly-ethylene-co-vinyl acetate polymer (Sigma-Aldrich, St. Louis, MO, USA). They were fixed to the skin using kinesiotape. The carbon nanotubes have a diameter of 1.6 mm and an electrical resistance of ~40 Ω/cm (for a concentration of 41 wt.%). The reference electrode of the NSS is a commercial Ag/AgCl electrode (Kendall 200, Covidien, Dublin, Ireland) positioned on the iliac crest.

The process control block is a custom-made system designed for sEMG recordings [35]. The acquisition module includes an ADS1294 (Texas Instrument, Dallas, TX, USA) low-power analog front end for biopotential measurements with 4 channels (sampling rate 2000 Hz, resolution 24 bits). Data are transmitted by a single-chip RF (radio frequency) transceiver, the nRF24L01 + 2.4 GHz (Sparkfun Electronics, Niwot, CO, USA). A base station with a receiver is connected to a host PC via a USB cable. The battery life of the NSS is approximately 8 h. The sEMG signals were recorded and processed with Matlab R2018b (MathWorks Inc., Natick, MA, USA).

#### 2.3.2. The Commercial Laboratory sEMG System (CSS)

The Delsys Trigno system [CSS], a wireless sEMG sensor system (4-electrode sensor block, 10 mm inter electrode spacing; packaged in a rigid rectangular box of 27 × 37 × 13 mm), with a dual on-board reference (Trigno system, Delsys Inc., Natick, MA, USA) was used as the reference instrument (sampling rate 1925.93 Hz, 16-bit resolution, Bluetooth transmission). The recorded signals were processed with the same Matlab code as for the NSS.

#### 2.3.3. Dynamometer

**Figure 2:**
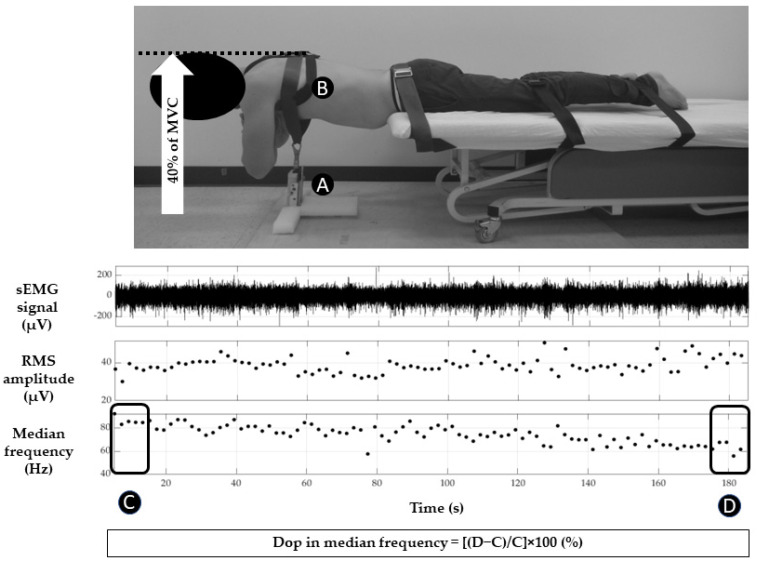
Modified Biering–Sorensen test at 40% of MVC: dynamometer (A), harness (B), example of one erector spinae sEMG recording with NSS (median frequency in Hz; Root Mean Square amplitude and EMG mean signal in µV) and time windows (C and D) used for the calculation of the drop in median frequency.

A dynamometer (Medup, Atlas Medic, St-Augustin-Desmaures, QC, Canada), secured to the floor and attached to a harness placed around the thorax, measured the back muscle force (N) generated by the participants (Figure 2). It allowed the measurement of maximal voluntary force (MVC) and provided force output feedback to the participants during the modified Biering–Sorensen test. It also confirmed the presence of fatigue (i.e., drop in MVC). The force signal was recorded at a sampling rate of 2000 Hz using the computer USB port and processed with Matlab R2018b.

### 2.4. Clinical and Force Tests Descriptions

For the maximal voluntary contraction and the modified Biering–Sorensen tests, the participants were positioned in a prone position (Figure 2). Three belts stabilized the participant’s body at the level of the pelvis, knee, and ankle joints, respectively. The trunk and upper body extended beyond the front edge of a treatment table, with the anterior superior iliac spine aligned with the bed’s edge.

#### 2.4.1. Force Test: Maximal Voluntary Contraction

Three MVCs were performed before and after the modified Biering–Sorensen test (see the test sequence in Figure 1). The MVC procedure involved aligning the trunk parallel to the floor and executing an isometric trunk extension with maximum effort for a duration of 3 s [37,38]. Five-second rest periods separated each MVC. Participants received verbal encouragement from the experimenter during each contraction. The average of the three sEMG amplitude plateaus on CSS and NSS was calculated. In addition, the back muscle force measured with the dynamometer was averaged for the three MVCs performed before and after the modified Biering–Sorensen test.

#### 2.4.2. Modified Biering–Sorensen Test

The Biering–Sorensen test is a clinical assessment of lower back fatigue, which involves measuring how long an individual can maintain a horizontal trunk position without support, while the rest of the body is secured to a treatment table [14,17,24]. In the modified Biering–Sorensen test, a submaximal (isometric) contraction against a cable is added to the maintenance of this position against gravity. This modified version of the original test has been used to differentiate between healthy individuals and those with low back pain [23,39,40]. In the current study, the modified Biering–Sorensen test was used to induce erector spinae muscle fatigue (see Figure 2). Healthy participants had to maintain a horizontal trunk position against 40% of their pre-test MVC, using the harness attached to the dynamometer fixed to the floor, for as long as possible until they placed their hands on the floor. Force and sEMG activity were recorded during the test. Real-time feedback to maintain the force was provided to the participants through two methods: (i) visual feedback using the dynamometer force output and (ii) verbal instructions from the experimenter.

#### 2.4.3. Flexion–Extension Test

**Figure 3:**
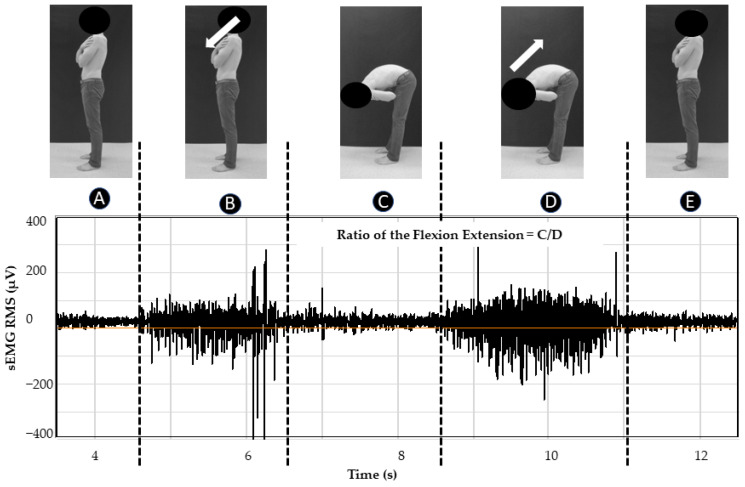
Movement phases of flexion–extension test: (A) initial rest period for 5 s, (B) spine flexion (leaning forward) in 5 s, (C) maintaining full spine flexion for 3 s, (D) spine extension from full flexion until the initial standing position was recovered in 5 s (E); example of one erector spinae sEMG RMS recording with NSS and windows used for the sEMG RMS ratio calculation (C, D).

The flexion–extension test was used to measure erector spinae muscle contraction and relaxation profiles (Figure 3). The participants completed five full range-of-motion spine flexions and extensions from a standing position. The task is composed of five phases (see details in Figure 3). Typically, healthy individuals exhibit an absence or minimal erector spinae EMG activity during full spine flexion (Figure 3, “C”) and substantial erector spinae EMG activity during spine extension from full flexion (Figure 3, “D”). Results from this test will be presented as the sEMG amplitude ratio of C/D (see Section 2.5.2).

### 2.5. sEMG

To assess NSS performance, erector spinae muscle activity was simultaneously recorded with the CSS and the NSS during both clinical tests. The two data sets were then synchronized off-line by cross-correlating the EMG signals from the NSS and the CSS and realigning the data based on the time delay of the peak of the correlation. This was done separately for each recording.

#### 2.5.1. Set-Up

sEMG sensors were installed after skin preparation (shaving and cleaning with alcohol) at the following spinal levels (see Figure 1): T12-L1 (NSS on the right side, CSS on the left side) and L4-L5 (NSS on the left side, CSS on the right side), 2 cm lateral from the spinous processes [41]. These two lumbar spinal levels were selected because they may exhibit different responses to low back pain during functional lower back movements [20].

#### 2.5.2. Signal Processing

Recorded signals from both systems were processed off-line using the same custom-written Matlab code. sEMG signals were first band-pass filtered (20–450 Hz) with a zero-lag 4th order Butterworth filter. sEMG signal amplitude (root-mean-square envelope of the EMG signal, RMS; 10 ms non-overlapping window) and the median frequency of the power spectrum (MDF) were calculated in 2 s (for the modified Biering–Sorensen test) and 0.5 s (for the flexion–extension test) windows.

The MDF of the power spectral density (%) was calculated from a squared Fast-Fourier Transform of the signal. The drop in median frequency was calculated as [42]:

$$\left(\frac{\left(average\;of\;last\;10\;s\;MDF\right)-\left(average\;of\;first\;10\;s\;MDF\right)}{average\;of\;first\;10\;s\;MDF}\right)*100$$

The sEMG RMS ratio (%) for the flexion–extension test was calculated as [43]:

$$\left(\frac{RMS\;sEMG\;amplitude\;full\;spine\;flexion}{RMS\;sEMG\;amplitude\;spine\;extension\;from\;full\;flexion}\right)*100$$

### 2.6. Statistical Analysis

Descriptive statistics (mean, standard deviation) were applied to the sociodemographic data, as well as to the lumbar extensor force measured with the dynamometer.

The chosen variables and statistical tests used for each objective of the study were as follows:

Objective 1—sensitivity of the NSS (non-parametric Wilcoxon matched paired tests):

Changes in median frequency during the modified Biering–Sorensen test (early vs. late MDF), as a measure of fatigue development.

Changes in RMS levels during the flexion–extension test (full flexion versus extension from full flexion), as a measure of muscle activation.

Objective 2—validity of the NSS in comparison to a reference instrument (CSS):

Validity of fatigue assessment during the modified Biering–Sorensen test (MDF) and validity of muscle activation pattern assessment during the flexion–extension test (RMS amplitude ratios): Wilcoxon matched paired tests between CSS and NSS.

Validity over time during the modified Biering–Sorensen test (raw MDF and RMS signals): cross-correlations between CSS and NSS signals. Cross-correlations are a well-established method to compare sEMG signals (spatial and frequential domain) [44,45]. The correlation values are considered as negligible (less than 0.3), low (between 0.3 and 0.5), moderate (between 0.5 and 0.70), good (between 0.70 and 0.9), or excellent (greater than 0.90) [45,46]. This assessment was only performed during the modified Biering–Sorensen test (larger number of data points per data collection).

Objective 3—Comparison of sEMG interpretations (MDF values, MDF%, and RMS amplitude) with dry skin versus conductive gel–electrode interfaces during the modified Biering–Sorensen test at L4-L5 left levels: Wilcoxon matched paired tests. In addition, associations between NSS and CSS variables, and between the different interfaces were tested using simple linear regressions.

## 3. Results

### 3.1. Participants

Twelve healthy participants (six females, 28.0 ± 3.3 years, 1.78 ± 0.11 m, 71.3 ± 15.5 kg, BMI 22 ± 2 kg/m^2^) were recruited for the study.

### 3.2. Recordings

Throughout data collection, most recordings from both systems were usable. However, upon conducting a thorough visual analysis of amplitude and frequency content, some recordings were excluded. The primary encountered issues were (1) a loss of skin–electrode contact, and (2) movement-induced electrical/mechanical artefacts occurring at the electrode cable–PCB interface. The number of usable recordings included in the analysis is indicated in parentheses throughout the text and in Table 1.

### 3.3. NSS Sensitivity

To assess the sensitivity of the NSS, we compared MDF-early (first 10 s into the test) to MDF-late (last 10 s into the test) during the modified Biering–Sorensen test for fatigue detection, and flexion vs. extension sEMG during the flexion–extension test for muscle activation pattern assessment.

#### 3.3.1. Fatigue Assessment (Change in MDF) 

As measured with the dynamometer, participants showed a significant reduction in lumbar extensor force after performing the modified Biering–Sorensen test (−104.67 ± 89.47 N; *p* < 0.05), confirming the development of fatigue in their lower back muscles.

**Figure 4:**
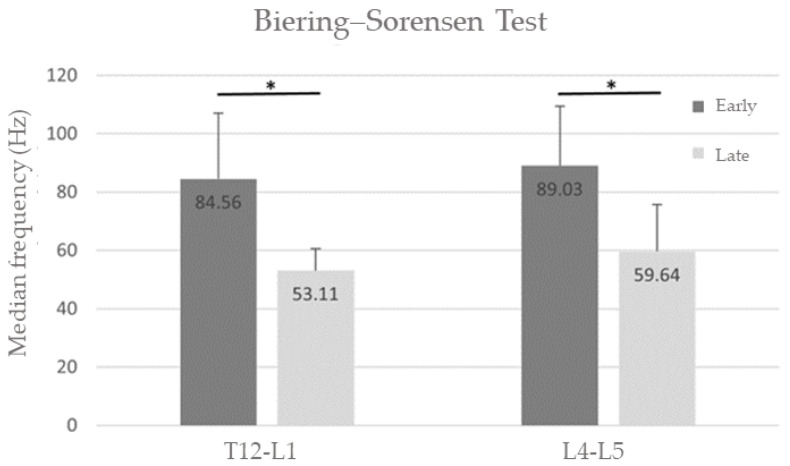
Group averages of the median frequency drop (MDF) of the erector spinae muscle power spectrum at the beginning (first 10 s—Early) and end (last 10 s—Late) of the Biering–Sorensen test. There was a significant drop in median frequency at both T12-L1 and L4-L5 spinal levels. * *p* < 0.05.

A statistically significant change in early versus late MDF (Table 1 and Figure 4) was observed both at T12-L1 (7/12 usable recordings, *p* < 0.05) and L4-L5 (9/12 usable recordings, *p* < 0.001).

#### 3.3.2. Muscle Activation Pattern Assessment (RMS Ratios)

A statistically significant change in full flexion versus extension from full flexion in RMS was observed during the flexion–extension test for both T12-L1 (8/12 usable recordings) and L4-L5 (8/12 usable recordings; *p* < 0.05; Table 1).

### 3.4. NSS Validity

To assess the validity of the NSS, it was compared to a commercial sEMG system (Delsys Trigno). Recordings were obtained simultaneously (see Methods).

#### 3.4.1. Validity of Fatigue Assessment (Change in MDF)

There were no statistically significant differences between the median frequency drops obtained from the NSS and the CSS, either at T12-L1 (*p* = 0.31) or at L4-L5 (*p* = 0.52) (Table 2). 

#### 3.4.2. Validity of the Muscle Activation Pattern (RMS Ratios)

**Figure 5:**
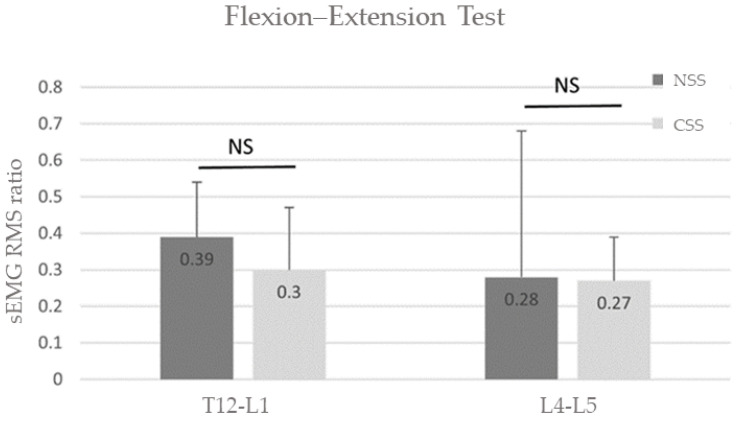
Group average sEMG RMS ratios for the NSS (black bars) and CSS (grey bars) measured during the flexion–extension test. There were no significant differences in measured ratios between the two recording systems.

There were no statistically significant differences in sEMG RMS ratios between the NSS and CSS for the flexion–extension test (T12-L1: *p* = 0.21; L4-L5: *p* = 0.67; Table 2 and Figure 5).

### 3.5. Validity over Time

To assess signal quality over time, we compared the raw signals obtained with the NSS with those from the CSS using cross-correlations during the modified Biering–Sorensen test. The mean group cross-correlations for sEMG MDF were 0.60 ± 0.27 at T12-L1 and 0.68 ± 0.32 at L4-L5. The cross-correlations for sEMG RMS amplitude were 0.53 ± 0.43 at T12-L1 and 0.62 ± 0.32 at L4-L5. Peak correlations were obtained at zero delay.

### 3.6. Impact of NSS Skin–Electrode Interface

**Figure 6:**
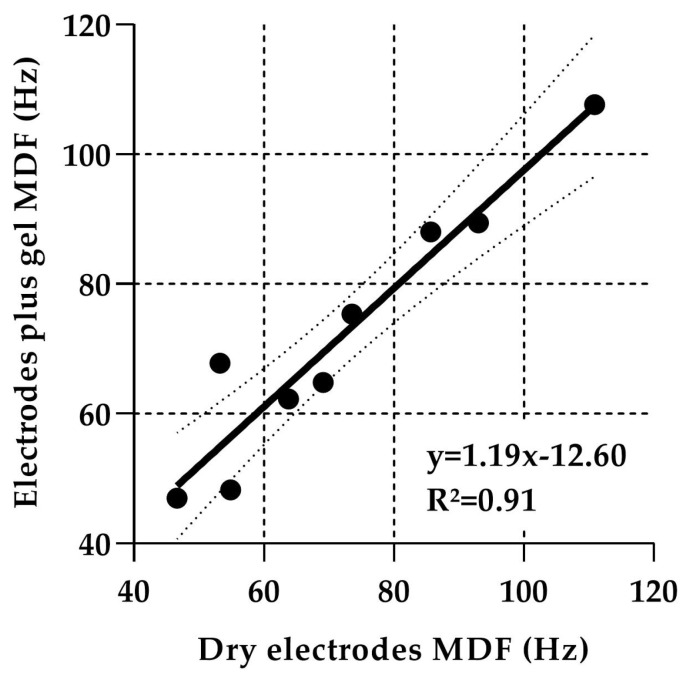
Linear regression analysis (y, black slope) with 95% confidence interval (dash lines), between NSS MDF (in Hz) measured with dry electrodes vs. electrodes plus gel. R^2^ is the variance explained by the model. Each circle represents one participant.

The modified Biering–Sorensen test was also used to examine the impact of dry skin versus gel–electrode interfaces at the L4-L5 lumbar spinal level. The mean group MDF was 72.29 ± 16.76 Hz (dry skin–electrode interface) versus 72.30 ± 20.93 Hz (gel–electrode interface), the MDF was −40.35 ± 16.05% versus −37.78 ± 19.87%, and finally RMS amplitude was 41.63 ± 9.25 µV versus 53.10 ± 2.70 µV. No statistically significant differences were observed between skin–electrode interfaces in terms of median frequency (*p* = 0.36), MDF (*p* = 0.16), or RMS amplitude (*p* = 0.25). In addition, NSS dry electrodes explain 91% of NSS electrodes plus gel variance on MDF (R^2^) with a simple linear relationship (Figure 6). Dry electrodes were as effective as electrodes plus gel to assess MDF.

## 4. Discussion

The primary objective of this study was to investigate the capabilities of the NSS for detecting electromyographic indicators of back muscle activation that may be altered in LBP during clinical testing. The NSS demonstrated sensitivity in measuring fatigue development during the modified Biering–Sorensen test and muscle activation patterns during the flexion–extension test.

When compared to the commercial system (objective 2), the NSS showed intra-participant validity as there were no statistically significant differences in fatigue and muscle activation patterns during the two clinical tests (drop in median frequency and RMS ratio). Additionally, it showed moderate cross-correlation regarding raw signal characteristics over time (amplitude and frequency) (0.5 ≤ cross-correlation) during the modified Biering–Sorensen test.

Finally, the use of dry electrodes at L4-L5 spinal level did not yield different sEMG interpretations (drop in median frequency and RMS ratio) compared to electrodes with conductive gel. 

The sensitivity of NSS in measuring the drop in median frequency as a measure of fatigue development and RMS ratios as a measure of muscle activation pattern is a first promising step. These results suggest that NSS could be a useful tool to better identify the presence/absence and anatomical location of low back pain-related sensorimotor alteration, and by doing so, to facilitate the identification of motor control alteration in LBP. Despite the rapidly emerging technology field of smart textiles, low-cost systems for measuring motor control deficits (i.e., fatigue and muscle activation patterns) [47,48] are not available. Only a few solutions exist for monitoring postural changes [49,50,51] that are the consequence of sensorimotor alteration. The NSS therefore presents a promising solution for LBP management by allowing simple measurement of changes in muscle activations during actual clinical tests.

The signal quality of the NSS compared to an expensive and more cumbersome laboratory CSS is also promising. The NSS allowed the same clinical test interpretations (e.g., drop in median frequency and RMS amplitude ratio), with a moderate cross-correlation of the signals over the fatigue test. The NSS seemed better at measuring sEMG frequency than amplitude. This capacity of measurement is a strength for muscle fatigue monitoring. In addition, the lumbar spinal level influenced NSS frequency signal quality due to higher MDF activity at a low lumbar spinal level, as noted in previous studies [20,25,52].

The NSS signal quality allows for a detailed analysis of task- and participant-specific muscle activation patterns. Furthermore, the flexibility of the NSS dry electrode sensors provided better signal quality (fewer movement artefacts) during movement than the CSS, thereby offering a positive alternative to CSS for clinical testing.

The secondary objective of this study was to quantify the impact of gel versus dry skin–electrode interfaces on NSS performance. As no statistically significant differences in MDF and RMS amplitude were recorded between the two systems, the dry electrode interface will greatly simplify the system’s use and uptake in the clinic. 

This study has several strengths. First, it illustrates the collaboration between engineers and clinicians to develop a new system as recommended in modern digital medicine [53]. Secondly, the NSS has a promising level of sensitivity, validity, flexibility, sewing, and washable capacities, making it a good prospect for textile integration.

This study also has some limitations. First, the interface between the multi-material polymer fibers and the process control block was challenging and produced artefacts rendering some recordings unsuitable for detailed spectral analysis. However, in the remaining recordings, sEMG signal quality was promising. The future textile integration of the sensor fiber will provide a good opportunity to improve these connections through fixation/sewing of the fiber and the electronics inside the garment. Secondly, the number of participants was too small to allow for a full validation of the system. However, this study was a very important proof of principle, showing the potential of the system. Future studies will be performed with more healthy participants and people with LBP.

## 5. Conclusions

The new sEMG system offers equivalent capacity to a more cumbersome and expensive commercial sEMG system at detecting early indicators of muscle fatigue and changes in muscle activations, two important indicators of sensorimotor problems associated with low back pain. In addition, the NSS has the added ability of being integrable in textile due to the small form factor and flexibility of its carbon nanotube–PEVA electrodes. The flexibility of the electrodes also helps in reducing movement artefact in the sEMG during the flexion–extension test, a test requiring a large range of movement of the participants’ lower back. Together, these elements make the NSS specifically suitable for reliably detecting electromyographic activation in low back muscles during clinical tests.

## Figures and Tables

**Table 1:** Sensitivity of the NSS in detecting muscle fatigue (changes in median frequency in hertz, Hz) and muscle activation patterns (changes in RMS in microvolts, µV). Assessments were made at thoracic (T) and lumbar (L) spinal levels. Results are presented as mean values ± standard deviation, based on the number of valid recordings.

Electrode Location	Fatigue Assessment (Modified Biering–Sorensen Test)			Muscle Activation Pattern Assessment (Flexion–Extension Test)		
	Early MDF (Hz)	Late MDF (Hz)	Early vs. Late	Full Flexion sEMG RMS (µV)	Extension from Full Flexion RMS (µV)	Full Flexion vs. Extension from Full Flexion
T12-L1	84.56 ± 22.44	53.11 ± 7.47	*p* = 0.018	22.21 ± 39.70	46.26 ± 63.08	*p* = 0.012
L4-L5	89.03 ± 20.46	59.64 ± 16.05	*p* = 0.008	8.69 ± 2.66	30.51 ± 22.25	*p* = 0.012

**Table 2:** Validity of the NSS in quantifying muscle fatigue (drop in median frequency) and muscle activation patterns (RMS ratio) as compared to the commercial sEMG system (CSS). Assessments were made at thoracic (T) and lumbar (L) spinal levels. Results are presented as mean values ± standard deviation, based on the number of valid recordings (*n*).

Electrode Location	EMG System	Fatigue Assessment (Modified Biering–Sorensen Test)		Muscle Activation Pattern Assessment (Flexion–Extension Test)	
		Drop in Median Frequency (%)	NSS vs. CSS	sEMG RMS Ratio	NSS vs. CSS
T12-L1	NSS	−46.89 ± 7.47 (*n* = 7/12)	*p* = 0.31	0.39 ± 0.15 (*n* = 8/12)	*p* = 0.21
	CSS	−43.79 ± 11.73 (*n* = 7/12)		0.30 ± 0.17 (*n* = 8/12)	
L4-L5	NSS	−40.35 ± 16.05 (*n* = 9/12)	*p* = 0.52	0.28 ± 0.40 (*n* = 8/12)	*p* = 0.67
	CSS	−37.27 ± 18.71 (*n* = 9/12)		0.27 ± 0.12 (*n* = 8/12)	

## Data Availability

Data are contained within the article.
